# Three-dimensional acetabular orientation during periacetabular osteotomy: a video analysis of acetabular rim position using an external fixator as navigation tool during reorientation procedure

**DOI:** 10.1007/s00402-020-03632-y

**Published:** 2020-10-24

**Authors:** Timo J. Schwarz, Günther Maderbacher, Franziska Leiss, Joachim Grifka, G. Heers, J. Matussek

**Affiliations:** 1 Department of Orthopedic Surgery, University Medical Center Regensburg, Kaiser-Karl-V.-Allee 3, 93077 Bad Abbach, Germany; 2 Department of Orthopedic Surgery and Arthroplasty, Vitos Orthopedic Clinic Kassel, Wilhelmshöher Allee 345, 34131 Kassel, Germany; 3 Departement of Pediatric Orthopedic Surgery, Klinikum Emil von Behring, Academic Teaching Hospital of Charite Berlin, Walterhöferstr. 11, 14165 Berlin, Germany

**Keywords:** Periacetabular osteotomy, Retroversion, Dysplasia, Hip surgery, Impingement, Navigation

## Abstract

**Introduction:**

Bernese periacetabular osteotomy is an effective procedure for treating acetabular dysplasia. However, limited visual control of the acetabular position during surgery may result in under- or overcorrection with residual dysplasia or femoroacetabular impingement. Thus, we wanted to find a simple method to control the effect of correction in the sagittal and coronal plane.

**Method:**

The acetabular coordinates are shown by two perpendicular tubes of an external fixator mounted onto a third tube that is fixed to the acetabular fragment with two Schanz screws. This method enables the isolated acetabular reorientation in the coronal, sagittal, and transverse plane. In a sawbone pelvis model, the acetabular rim is marked with a copper wire and a silicon adherent. To show the radiographic effect on acetabular parameters and the rim position, we visualized correction in the coronal and sagittal plane under fluoroscopic control.

**Results:**

Lateral rotation of the acetabular fragment had the highest impact on radiographic lateral coverage of the femoral head. But also ventral coverage increased during isolated lateral rotation. Anterior rotation showed almost no effect on lateral coverage and just a little effect on ventral coverage but caused severe total acetabular retroversion.

**Conclusion:**

Three-dimensional control of the acetabular orientation during periacetabular osteotomy is important to avoid over- and under-correction. Isolated lateral rotation of the acetabular fragment should be the predominant direction of correction during periacetabular osteotomy. Ambitious anterior correction may be the main source for severe acetabular retroversion following periacetabular osteotomy.

**Electronic supplementary material:**

The online version of this article (10.1007/s00402-020-03632-y) contains supplementary material, which is available to authorized users.

## Introduction

Bernese periacetabular osteotomy (PAO) for hip dysplasia—a method popularized by the work of Ganz et al. [1]—has shown to be an effective procedure to improve acetabular coverage, reduce pain, and improve hip function in several mid- to long-term studies [2–6]. Despite the intensity of surgical intervention, short-term results after 1 year have shown both improvements in patients’ physical activity levels and decreases in pain [7]. In a subpopulation of athletes, Heyworth et al. reported good results and a median return-to-play period of 9 months after PAO in 80% of patients [8]. The first long-term results after 30 years show good results without any progression in arthritis or conversion to THA for one third of PAO procedures [9].

Poor results after PAO are either due to patient selection or inadequate acetabular reorientation. Patient-specific factors associated with failure after pelvic osteotomy are advanced age, increased radiographic joint degeneration, and intensified preoperative pain [3, 5, 10–12]. Acetabular under- and overcorrection may cause pain due to residual hip dysplasia or femoroacetabular impingement [3–5, 10, 13]. Acetabular reorientation is challenging, and even experienced surgeons have reported inadequate postoperative radiographic coverage (lateral CE angle) in approximately one fifth of the patients [14]. But particularly malorientation such as total acetabular retroversion causes postoperative impingement, a restricted range of movement, and a positive Drehmann’s sign [15]. Some of these patients even required revision surgery, such as revision PAO or difficult total hip arthroplasty.

For this reason, the aim of this investigation was to show potential sources of error during the reorientation procedure and to describe method that allows visual control of the acetabular position. Therefore, we used an external fixator as an analogous navigator to show the acetabular coordinates and to simulate different directions of correction under fluoroscopic control in a sawbone pelvis model. This study is not meant to be a publication of a novel operative technique even though the external fixator is suitable for intraoperative application.

## Method

**Fig. 1:**
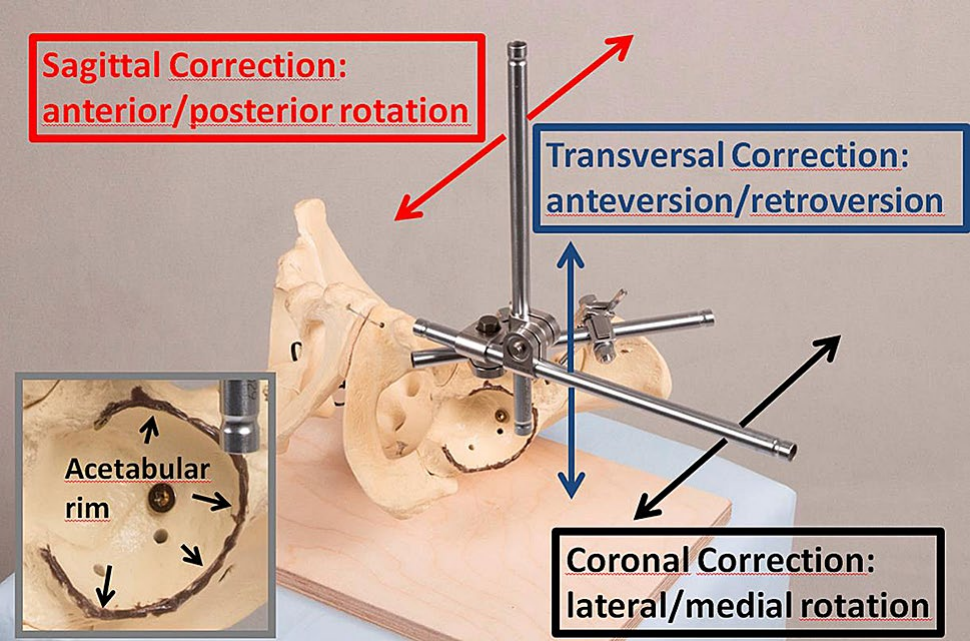
A sawbone pelvis model with the external fixator mounted onto the acetabular fragment. The red, blue, and black arrows show the direction of correction in the sagittal, transversal, and coronal plane. The acetabular rim is marked with a copper wire and a silicon adherent (left)

A sawbone pelvis model was fixed to a radiolucent board in supine position. To be able to follow the acetabular rim position by fluoroscopy during the correction procedure, a thin copper wire was fixed to the rim with a silicone adhesive (Fig. 1). The acetabular fragment was detached from the pelvis by four saw cuts analogous to the PAO. An external fixator was mounted with two Schanz screws located superiorly and inferiorly to the anterior inferior iliac spine. Under anatomic reduction, two perpendicular tubes were connected to the external fixator to show the acetabular coordinate system in supine position (Fig. 1). Pulling on tubes of the external fixator in the coronal, sagittal, and transversal plane enabled the investigation of defined motions of the acetabular fragment: lateral/medial rotation, anterior/posterior rotation, and anteversion/retroversion. Particularly lateral and anterior rotations are common directions of correction to improve lateral and ventral coverage of the hip. To show the radiographic effect of these two directions of correction, the correction procedure (lateral and anterior rotation of the acetabular fragment around the femoral head) was simultaneously filmed with fluoroscopic visualization. To show the effect on lateral and ventral coverage, fluoroscopy was once conducted in anteroposterior X-ray view and once in faux profil X-ray view. Thus, we analyzed pre- and post-interventional acetabular rim position, acetabular version, and anterior and lateral coverage of the femoral head by means of four video clips (anterior and lateral rotation under anteroposterior and faux profil X-ray view).

## Results

**Fig. 2:**
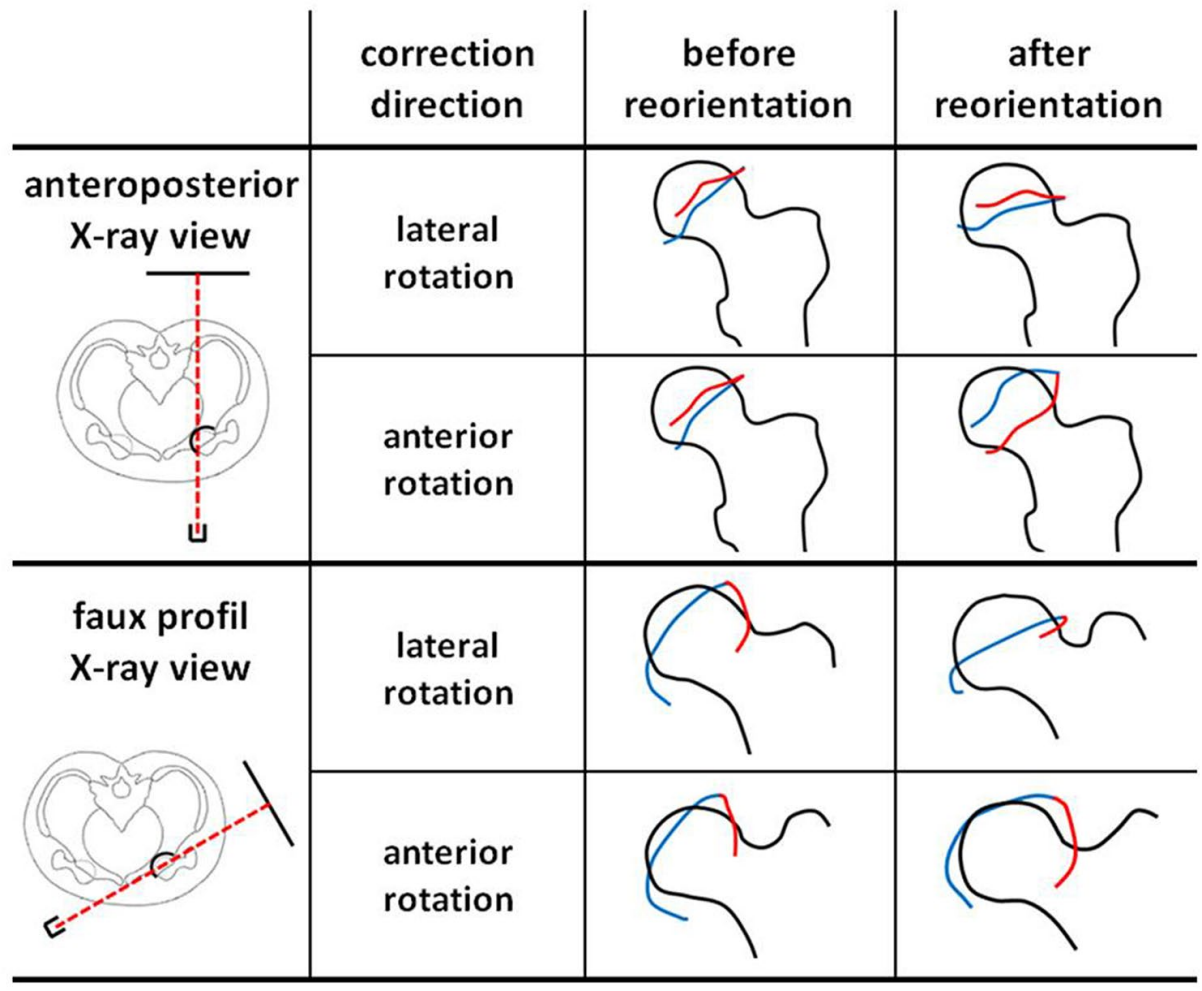
Analysis of correction directions (lateral rotation and anterior rotation) under anteroposterior (above) and faux profil (below) X-ray view. The anterior (red) and posterior (blue) acetabular rim is shown before and after acetabular reorientation. These rim positions correspond to the radiographic positions at the beginning and end of the corresponding video clips

In each of the four video clips, the femoral head position as well as the anterior and posterior acetabular rims were marked before and after the correction procedure, visualizing the effect of acetabular correction as shown in Fig. 2.

Analyses of correction procedures under anteroposterior fluoroscopic view:

Correction in the coronal plane (lateral rotation) under anteroposterior fluoroscopic view is shown in the first video clip (Clip1_APview_lateralrotation.mp4) and Fig. 2. Compared to the initial X-ray image, the acetabulum shows identical configuration. This means the anterior and posterior acetabular rims stay in the same anatomic position without any change in acetabular version. We termed this consistency of configuration ‘radiographic excision effect’, because it resembles an X-ray image in which someone excised the acetabulum and rotated it clock- (left hip) or counterclockwise (right hip). Analyzing the lateral coverage in the AP radiograph, correction in the coronal plane did immediately affect the LCE angle. Thus, there is a direct correlation between angular lateral rotation and the LCE angle.

Correction in the sagittal plane (anterior rotation) under anteroposterior fluoroscopic view is shown in the second video clip (Clip2_APview_anteriorrotation.mp4) and Fig. 2. Anterior rotation of the acetabulum caused a lateral shift of the anterior rim and a medial shift of the dorsal rim. Thus, acetabular version changed significantly leading to total acetabular retroversion, which may cause severe ventral femoroacetabular impingement. No relevant change in lateral coverage was found when comparing the CE angle before and after the correction procedure.

Analyses of correction procedures under faux profil fluoroscopic view:

Radiological faux profil view is suitable for evaluating anterior coverage (VCA angle) and rim positions in the sagittal plane. The third video clip (Clip3_FauxProfil_lateralrotation.mp4) shows lateral rotation of the acetabulum (coronal plane correction) under faux profil view. The anterior rim did not change significantly, but the posterior rim was shifted ventrally, causing the anterior shift of the anterior edge. Thus, anterior coverage (VCA angle) was significantly increased. Finally, in the fourth video clip (Clip4_FauxProfil_anteriorrotation.mp4), acetabular anterior rotation (sagittal correction) is shown under fluoroscopic view. The anterior rim was shifted ventrally, and the posterior rim dorsally. Thus, there was only a slight ventral shift of the anterior edge, increasing ventral coverage. An anterior rim shifted to the direction of the femoral neck bears a high risk of femoroacetabular impingement.

**Fig. 3:**
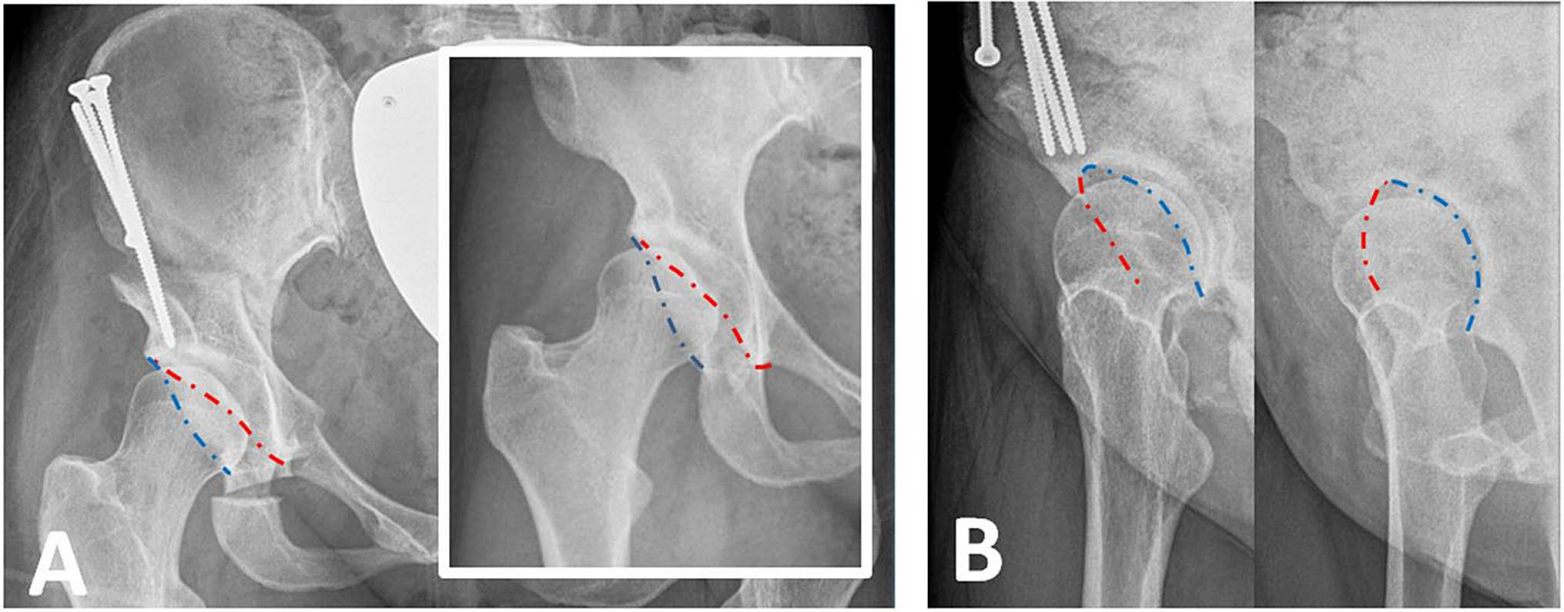
Pre and postoperative X-ray of a female dysplasia following periacetabular osteotomy in anteroposterior view (**a**) and faux profile view (**b**). The dashed line shows the anterior (red) and posterior (blue) acetabular rim

Clinical concordance with the above mentioned findings is reproducible in postoperative X-rays following periacetabular osteotomy. In Fig. 3, pre- and postoperative X-rays of a female dysplasia are shown in anteroposterior and faux profil view. The CE angle of 14° was corrected to a CE angle of 30°. We used the external fixator as an analogous navigator to perform isolated lateral correction. The anterior and posterior acetabular rims stayed in the same anatomic position without any change in acetabular version. Although no anterior rotation was performed, in faux profil X-ray view an improved anterior coverage from VCA angle from 14° to 28° was measured.

## Discussion

The success or failure of periacetabular osteotomy is strongly determined by the correct postoperative acetabular cup position. In this study, we described a method for improving spatial control of the acetabular cup position during reorientation in three planes (coronal, sagittal, and transversal plane). To show the effect on femoral coverage and acetabular orientation, we analyzed correction videos in different X-ray projections. In these videos, the correction procedure in the coronal plane (lateral rotation) showed excellent improvement of lateral and—surprisingly—also of ventral coverage (LCE angle and VCA angle) without any changes in acetabular version. Correction in the sagittal plane (anterior rotation) showed severe changes in acetabular version, causing total acetabular retroversion. No improvement in lateral coverage (LCE-angle) was measured, and the effect on ventral coverage was even less than that during correction in the coronal plane (lateral rotation).

Anterior and lateral coverage increase simultaneously when the acetabular fragment is rotated laterally. Thus, to avoid retroversion or femoroacetabular impingement, anterior rotation should be performed very carefully, especially if the preoperative X-ray image already shows low acetabular anteversion or cranial acetabular retroversion (crossing sign). Surgeons should take this finding into consideration when planning periacetabular osteotomy.

Because we used a normal sawbone pelvis model without dysplastic acetabular configuration, general conclusions from our video analysis are limited. But our findings are in line with the recommendations of two studies that virtually investigated the optimal postoperative acetabular position in dysplastic hips [16, 17]. 3D finite elements models were generated from CT scans of dysplastic hips, and contact area, pressure, and Mises stress were calculated after virtual periacetabular osteotomy at different correction angles. In three dysplastic hips (CE angles of 10°, 13°, 20°) investigated by Arturo Chavez, optimal correction was achieved by coronal correction only, and no anterior rotation was needed [17]. Similar results were achieved by Wang et al. [16]. During reorientation for optimal correction in three hips with CE angles of 19°, 7°, and − 7°, optimal position was primarily achieved by lateral rotation of 17°, 25°, and 30° in the coronal plane, respectively. In two cases of severe dysplasia (CE angle − 7° and 7°), the contact area was additionally improved by anterior rotation in the sagittal plane by 10° and 5°. But the authors raised concerns regarding anterior correction because it may cause anterior femoroacetabular impingement and hip joint instability by aggravating deficiency in posterior coverage. For this reason, surgeons should be careful with regard to anterior rotation [16].

Because the acetabular cup position cannot be made visible during PAO, the intraoperative implementation of the optimal, preoperatively planned correction may be difficult. Not only anterior and lateral rotation must be controlled but also the axial rotation (anteversion/retroversion) of the acetabular fragment. To improve spatial control during reorientation, we apply the external fixator to the acetabular fragment during every periacetabular osteotomy. Thus, the acetabular coordinates are shown, and correction can be controlled visually in the coronal, sagittal, and transversal plane.

Tracking the correction of the acetabular fragment during PAO is challenging and affords intraoperative radiographs or fluoroscopy. For this reason, different analogous [18], and digital [19–21] techniques have been developed to improve the accuracy of acetabular reorientation. By developing a measuring device for intraoperative assessment of the acetabular index (AI) and the center edge angle (CE), Troelsen et al. have provided a useful tool for controlling these parameters by means of intraoperative fluoroscopy. But this tool does not provide 3D control of the acetabular fragment, thus control of acetabular version and anterior rotation is not possible. Promising approaches for intraoperative digital navigation were provided by two studies using optical navigation methods [19, 20]. In sawbone models, preoperative planning was successfully implemented in acetabular cup reorientation with high accuracy. Both methods take pelvic tilt into consideration and use the anterior pelvis plane as a reference for intraoperative navigation. Whereas Liu et al. considered three-motion components in the sagittal, coronal, and transversal plane, Pflugi et al. measured acetabular orientation by means of its anteversion and inclination. Assessment of acetabular orientation by means of anteversion and inclination is well known from total hip arthroplasty [22–24]. Description of spatial orientation of a 3D object (acetabular cup) using 2 parameters only (anteversion and inclination) is just possible if the object has an axis of symmetry. During THA, normal acetabular cups are configured symmetrically, thus rotation along their axis of aperture can be neglected. In contrast, native acetabulum is not configured symmetrically, thus a third spatial parameter must be considered. For this reason, we recommend tracking acetabular reorientation in the sagittal, coronal, and transversal plane. Regarding Murray’s definitions of cup orientation, acetabular reorientation in 3 planes changes its operative anteversion (sagittal correction), anatomical anteversion (transversal correction), and radiographic inclination (coronal correction). In our method, we use the external fixator to follow the acetabular coordinates during reorientation, enabling the tracking of the correction in three planes. Since reorientation is considerably relieved, we have used the external fixator for all PAO procedures since its introduction into our surgical process in 2018. Although the application of the external fixator takes some time, the time of surgery is not significantly lengthened because of faster correct reorientation, fewer intraoperative X-rays images, and better control of the acetabular fragment during the following surgical steps such as screw fixation.

We hope that the findings of this study will contribute to the development of future digital navigation methods for PAO. Thus, preoperative planning will be more exactly realized during surgery. The high importance of controlling reorientation in 3 planes, as mentioned above, calls for the digital visualization of fragment correction in the sagittal, coronal, and transversal plane.

## Conclusion

Three-dimensional control of the acetabular orientation during periacetabular osteotomy is important to avoid over- and under-correction as well as acetabular retroversion. In this article, we describe a method for visualization of acetabular coordinates using an external fixator enabling us the analysis of isolated anterior and lateral correction. Isolated lateral rotation of the acetabular fragment should be the predominant direction of correction during periacetabular osteotomy. Ambitious anterior correction may be the main source for severe acetabular retroversion following periacetabular osteotomy.

## Electronic supplementary material

Below is the link to the electronic supplementary material.

Supplementary file1 (MP4 62405 kb)

Supplementary file2 (MP4 73926 kb)

Supplementary file3 (MP4 55262 kb)

Supplementary file4 (MP4 52501 kb)

## References

[1] Ganz R, Klaue K, Vinh TS (1988). A new periacetabular osteotomy for the treatment of hip dysplasias. Technique and preliminary results. Clin Orthop Relat Res.

[2] Clohisy JC, Barrett SE, Gordon JE (2005). Periacetabular osteotomy for the treatment of severe acetabular dysplasia. J Bone Joint Surg Am.

[3] Albers CE, Steppacher SD, Ganz R (2013). Impingement adversely affects 10-year survivorship after periacetabular osteotomy for DDH. Clin Orthop Relat Res.

[4] Dahl LB, Dengso K, Bang-Christiansen K (2014). Clinical and radiological outcome after periacetabular osteotomy: a cross-sectional study of 127 hips operated on from 1999–2008. Hip Int.

[5] Steppacher SD, Tannast M, Ganz R (2008). Mean 20-year follow up of Bernese periacetabular osteotomy. Clin Orthop Relat Res.

[6] Siebenrock KA, Scholl E, Lottenbach M (1999). Bernese periacetabular osteotomy. Clin Orthop Relat Res.

[7] Novais EN, Heyworth B, Murray K (2013). Physical activity level improves after periacetabular osteotomy for the treatment of symptomatic hip dysplasia. Clin Orthop Relat Res.

[8] Heyworth BE, Novais EN, Murray K (2016). Return to play after periacetabular osteotomy for treatment of acetabular dysplasia in adolescent and young adult athletes. Am J Sports Med.

[9] Lerch TD, Steppacher SD, Liechti EF (2017). One-third of hips after periacetabular osteotomy survive 30 years with good clinical results, no progression of arthritis, or conversion to THA. Clin Orthop Relat Res.

[10] Hartig-Andreasen C, Troelsen A, Thillemann TM (2012). What factors predict failure 4 to 12 years after periacetabular osteotomy?. Clin Orthop Relat Res.

[11] Kralj M, Mavcic B, Antolic V (2005). The Bernese periacetabular osteotomy: clinical, radiographic and mechanical 7–15-year follow-up of 26 hips. Acta Orthop.

[12] van Hellemondt GG, Sonneveld H, Schreuder MHE (2005). Triple osteotomy of the pelvis for acetabular dysplasia: results at a mean follow-up of 15 years. J Bone Jt Surg Br.

[13] Grammatopoulos G, Wales J, Kothari A (2016). What is the early/mid-term survivorship and functional outcome after Bernese periacetabular osteotomy in a pediatric surgeon practice?. Clin Orthop Relat Res.

[14] Novais EN, Duncan S, Nepple J (2017). Do radiographic parameters of dysplasia improve to normal ranges after Bernese periacetabular osteotomy?. Clin Orthop Relat Res.

[15] Tannast M, Pfander G, Steppacher SD (2013). Total acetabular retroversion following pelvic osteotomy: presentation, management, and outcome. Hip Int.

[16] Wang X, Peng J, Li D (2016). Does the optimal position of the acetabular fragment should be within the radiological normal range for all developmental dysplasia of the hip? A patient-specific finite element analysis. J Orthop Surg Res.

[17] Chavez A (2016) Mechanical analysis of a virtual Ganz periacetabular osteotomy in patients suffering hip malformations by using finite element analysis: a Thesis submitted to the University of Manchester for the degree of Doctor of Philosophy in the Faculty of Engineering and Physical Sciences. School of mechanical, aerospace and civil engineering

[18] Troelsen A, Elmengaard B, Romer L (2008). Reliable angle assessment during periacetabular osteotomy with a novel device. Clin Orthop Relat Res.

[19] Pflugi S, Liu L, Ecker TM (2016). A cost-effective surgical navigation solution for periacetabular osteotomy (PAO) surgery. Int J Comput Assist Radiol Surg.

[20] Liu L, Siebenrock K, Nolte L-P (2018). Computer-assisted planning, simulation, and navigation system for periacetabular osteotomy. Adv Exp Med Biol.

[21] Jager M, Westhoff B, Wild A (2004). Computer-assisted periacetabular triple osteotomy for treatment of dysplasia of the hip (Computernavigierte Dreifach-Osteotomie des Beckens zur Behandlung der Huftgelenkdysplasie). Z Orthop Ihre Grenzgeb.

[22] Murray DW (1993). The definition and measurement of acetabular orientation. J Bone Jt Surg Br.

[23] Schwarz T, Weber M, Worner M (2017). Central X-ray beam correction of radiographic acetabular cup measurement after THA: an experimental study. Int J Comput Assist Radiol Surg.

[24] Schwarz TJ, Weber M, Dornia C (2017). Correction of pelvic tilt and pelvic rotation in cup measurement after THA—an experimental study (Korrektur der Beckenverkippung und der Beckenverdrehung bei der Pfannenmessung nach Huft-TEP-Versorgungen - Eine experimentelle Studie). Rofo.

